# Homeopathy, fundamentalism, and the memory of water

**DOI:** 10.3747/co.2007.161

**Published:** 2007-12

**Authors:** Lionel R. Milgrom

**Affiliations:** 17, Skardu Road, London NW2 3ES, United Kingdom E-mail: lionel.milgrom@hotmail.com

The Editor,

Current Oncology

October 12, 2007

**Re:** Homeopathy: does a teaspoon of honey help the medicine go down? Sagar SM. *Curr Oncol* 2007; 14:126–7.

Sir,

Pointing out the obvious (i.e., that seeking homeopathic treatment for cancer prior to early conventional diagnosis could be dangerous, and claiming efficacy for homeopathy’s ultra-highly diluted remedies runs counter to Avogadro’s hypothesis) does not exonerate Dr. Sagar’s editorial of grossly misrepresenting the Memory of Water (mow) hypothesis 1.

This compounds an unwarranted slur not only on homeopathy, those who practice it, and the millions around the world who benefit from it, but the many scientists researching mow. For his description of mow simply ignores recent published research from the materials, physical, and biochemical sciences 1–6. In addition, the editorial evinces the usual evangelical faith in the “gold standard” drug-testing procedure, the double-blind randomized-controlled trial (dbrct), and its presumed infallibility at providing incontestable evidence for or against the efficacy of homeopathy, or indeed any therapeutic modality 7.

First, Hahnemann never suggested water might be able to “remember” anything: mow is a 20th-century epithet. Second, mow has nothing whatever to do with “subatomic fields”: this would mean invoking the kind of particle–particle interactions more familiar from high-energy physics experiments! Rather, mow is a supramolecular phenomenon involving trillions of water molecules 8, dynamically interrelated and correlated by well-characterized intermolecular forces (for example, non-static hydrogen bonding, van de Waal interactions, and so on), which would no doubt survive interaction with the –OH groups of lactose. There is now published literature (some of which I include here) in reputable journals supporting the kind of dynamic water “structures” alluded to by mow 1–5. Its take-home message is that it is water **structure,** not content, that is important 2.

We are still left with the problem of how mow might lead to cure of the patient. I would agree that there is, as yet, much speculation but little research in this area to repudiate. Assuming it were ever to get the proper funding, however, such research would require much closer collaboration between the physical and biomedical sciences, and is perhaps a timely reminder of the intellectual parochialism that is a perennial feature of the latter.

As for the dbrct, its methodology assumes implicitly that a therapeutic intervention and the context in which it is given may be considered in complete isolation from one another. Thus, therapeutic modality (in homeopathy, the remedy; in acupuncture, the needle) provides efficacy, while the extended interview provides context. Ultimately, it is this separation of therapy and context that justifies testing against a “placebo,” one of the least understood concepts in biomedicine.

Of course, all this fits neatly into the biomedical paradigm and its assertion of a purely molecular basis to disease. In reality, problems arise for the dbrct immediately the separation of therapy and context is realised for what it is: an arithmetic convenience allowing measurements to be made, statistics to be gathered, and inferences to be drawn. Patient individuality [something long accepted—in addition to disease/ remedy similarity—in homeopathy and in complementary and alternative medicine (cam), but generally of far lesser importance in conventional biomedicine] means that therapy and context have to be seen as intimately correlated. Under these circumstances, a fundamentalist approach to the principles of the dbrct turns it into a blunt instrument that breaks this correlation, effectively destroying the very therapeutic effect it is trying to investigate 7. It is not surprising, therefore, that over time, dbrcts of homeopathy and other cams deliver at best equivocal results or reductions in effect sizes, or both.

Perhaps the dbrct is not the “gold standard” of research quality after all, but the scientific equivalent of Nelson putting a telescope to his blind eye ... and far too coarse an experimental procedure. It can similarly be argued that the dbrct isn’t always appropriate for investigating conventional medicine either 9. For, in real-life circumstances, no therapeutic intervention—conventional medicine included—is ever practiced according to the fundamentalist strictures of the dbrct. Its blindness (methodologic and sometimes deliberate) to the toxic side effects of certain drugs might help explain the dangers posed by some recent high-profile pharmaceuticals—for example, Seroxat, Vioxx, statins, and so on. Interestingly, a recent report from the House of Commons Public Accounts Committee in the United Kingdom concluded that at least 2.68 million people were harmed during 2006 by conventional medical intervention, representing a staggering 4.5% of the U.K. population 10. At least homeopathy isn’t this dangerous, and Hippocrates, no doubt, is turning somersaults in his grave. Not surprisingly therefore, there is a growing challenge to evidence-based medicine (the s) edifice being constructed on dbrct 11 coming from within conventional medicine itself 12,13.

To conclude, perhaps it is sufficient to recognise (a) that the current biomedical paradigm does not hold all the answers to sickness and health; (b) certain therapeutic modalities like homeopathy, while conventionally incomprehensible, are used, approved, and trusted by millions of people worldwide; and (c) the public are not stupid: they are highly unlikely to visit a homeopath if fixing a broken leg, triple bypass surgery, or indeed early cancer diagnosis is required. They might, however, wish to use appropriate homeopathic/cam treatment to help recovery, regardless of what the good doctors of physik say about the plausibility or otherwise of a particular therapy.

Thus, the humane way forward is to increase patient choice via more rational health care systems in which cam therapies such as homeopathy are available, cost effective, and integrated into primary healthcare. For it is patients who matter, and they will choose what is best for them. No one has a monopoly on truth, and ultimately, all healing, be it homeopathy, witch-doctoring, or even conventional medicine, begins between two consenting beings. Perhaps in our saner moments, we should try to remember this, because it could go a long way in helping all health practitioners work together toward the same goal: the best we can do for our patients.
